# Analysis of satisfaction patient and increased dermis thickness by medical evaluation and USG by Rennova Elleva in the treatment of sagging skin on the inner part of the arms

**DOI:** 10.1002/ski2.163

**Published:** 2022-09-25

**Authors:** Marisa Gonzaga da Cunha, Felipe Mattos Ferregutti, Amanda Cristina Bernardo, Pedro Ivo Romani, Carolina Nascimento, Rogério Ruiz

**Affiliations:** ^1^ Department of Dermatology Faculdade de Medicina do ABC São Paulo Brazil; ^2^ Department of Plastic Surgery Pontifícia Universidade Católica de São Paulo Faculdade de Medicina de Sorocaba São Paulo Brazil; ^3^ Universidade Federal de Goias Goiania Brazil

## Abstract

**Introduction:**

The flaccidity of the skin is provoked by changes caused by the chronological ageing of the skin, such as epidermal, dermal, and hypodermic thinning, which, in turn, are aggravated by photo ageing and by several other factors such as, for example, limited diets low in proteins, rapid weight loss and low BMI, liposuction, post‐pregnancy and stretch marks, which facilitate the loss of skin elasticity, even in young patients. Its treatment remains a major therapeutic challenge, as there are few procedures designed to effectively improve it.

**Objective:**

To evaluate the satisfaction patient and increased dermis of Rennova Elleva in the treatment of cutaneous flaccidity of the inner part of the arms.

**Methods:**

Twenty six women aged between 31 and 60 years old complaining of skin flaccidity in the medial area of the arms, with a severity score between: 1 (11 patients), 2 (10 patients) and 3 (5 patients) according to the scale *Investigator Assessment Skin Laxity Scoring System (IASLSS)*, were treated with two applications of poly‐L‐lactic acid (PLLA) with intervals of 45 days between them.

**Results:**

Forty five days after the second application of PLLA (D90), all patients presented a moderate to high degree of satisfaction with the treatment, with good tolerability and no adverse effects. Improvement was confirmed by high‐resolution ultrasound with an increase in dermal thickness between 23% and 70% (average value: 46%).

**Discussion:**

Rennova Elleva® PLLA promoted an improvement in skin flaccidity and an increase in dermal thickness with good to excellent tolerability by patients.

**Conclusion:**

The new presentation of PLLA proved to be effective and safe for the treatment of skin flaccidity.

## Graphical abstract

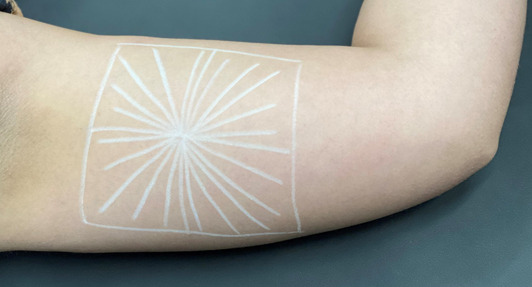

The flaccidity of the skin is provoked by changes caused by the chronological ageing of the skin, such as epidermal, dermal, and hypodermic. It was analysis of patient satisfaction and increased dermis thickness of Rennova Elleva in the treatment of cutaneous flaccidity of the inner part of the arms, promoting an improvement in skin flaccidity and an increase in dermal thickness with good to excellent tolerability by patients. The new presentation of poly–L–lactic acid proved to be effective and safe for the treatment of skin flaccidity.

## 1 INTRODUCTION

Beauty and attractiveness are important sociocultural concepts that tend to dictate how individuals are judged and treated. With the advancement of medicine and increased longevity, the demand for aesthetic procedures in different age groups and socioeconomic classes is increasing.

For the treatment of facial ageing there is a wide variety of products that open up a series of possibilities for combinations that will have synergistic effects, covering most of the needs of each patient. In addition to improving the appearance of the face, the search for the perfect body has become a concern for a large part of the population, especially women. However, the treatment of sagging skin remains a major therapeutic challenge, as there are few procedures aimed at effectively improving it.

The changes caused by the chronological ageing of the skin are the result of the normal physiological process and are observed due epidermal, dermal and hypodermic thinning which, in turn, are aggravated by photoaging. In the body, in addition to ageing, several factors contribute to the appearance or worsening of sagging skin, such as limited diets low in proteins, quick weight loss and low BMI, liposuction, pregnancy and stretch marks, which facilitate the loss of skin elasticity, even in young patients.^1^

Women are more affected by flaccidity for several reasons: the dermis in women is thinner; in men, the fibrous septa in the hypodermis are smaller and arranged in oblique planes with small lobes of fat, while in women the lobules are larger and with parallel septa,^2^ offering less resistance to stretching^3^; in women, the reduction in serum oestrogen levels observed from the climacteric period leads to even more skin extensibility and less elasticity^2^, ^4^; as a consequence, clinically, the skin appears thinner, drier and more flaccid; the woman suffers more situations during her life that cause skin stretching, such as pregnancy. Flaccidity can also be aggravated by lifestyle habits, such as smoking, limited diets, and weight variations.

Injectable collagen stimulators are widely used to ensure skin and soft tissue restructuring. Its use was driven by the fact that it guarantees relatively simple and quick application, along with safety and effectiveness.^4^

Injectable poly‐L‐lactic acid (PLLA) has been applied as a cosmetic filler since 1999 to correct skin volumetric losses caused by ageing in a gradual, progressive, and prolonged manner, promoting natural and harmonious results with low risk of adverse effects. It is a polymer of heavy molecule (140kD), part of the family of α‐hydroxy acids, derived from lactic acid, presented in the form of spherical particles with a smooth surface, measuring between 40 and 63 μm in diameter, dispersed as lyophilized powder in a sterilized bottle, added to sodium carboxymethyl cellulose and non‐pyrogenic mannitol. For application to be made, it must be reconstituted in sterilized distilled water for injection, which will be absorbed in 24–48 h after application.^5^, ^6^ A distinctive feature of the Rennova Elleva® product is the more uniform granulometric distribution of the particles and the lyophilization process that facilitates their reconstitution.^7^

Two hours after implantation of PLLA in the deep reticular dermis or superficial hypodermis, an initial mild inflammatory response begins with a reaction to foreign bodies in which macrophages fuse into giant cells to try to phagocytose the particles. Oedema appears to facilitate cell migration. Between seven and 10 days after implant introduction, macrophages secrete chemotactic and growth factors to initiate fibroblast attraction and the proliferative phase of reconstruction. Fibroblasts secrete extracellular matrix components, initially collagen type I and, to a lesser extent, type III collagen.^6^

Therefore, the fibroblasts isolate each implant particle with a fibrous collagen capsule, with substantial deposition of collagen type II close to the particles and disposition of collagen type I in the periphery of the encapsulated PLLA, forming a mature vascularized fibrous tissue.^6^ Thus, neocollagenesis and consequent increase in dermal thickness is obtained by the marked activity and fibroblast proliferation around each particle, which will be accompanied by the degradation of PLLA, through hydrolysis, into lactic acid monomers with no indication of an acute inflammatory reaction.^4^, ^5^, ^6^

The objective of this study was to evaluate the clinical reaction and the safety of the treatment of cutaneous flaccidity of the internal areas of the arms using a new presentation of PLLA (Rennova Elleva®), approved by ANVISA for commercialization in Brazil since the end of 2020. The study was performed and conducted in accordance with Good Clinical Practices.

## 2 METHODS

Twenty‐six women aged between 31 and 60 years (average age 46 years), skin types between II and IV, complaining of skin flaccidity in the medial area of the arms were treated.

**Inclusion criteria**: female gender with at least score I of skin flaccidity in the medial area of the arms, according to the *Investigator Assessment Skin Laxity Scoring System* (IASLSS) by Blyumin‐Karasik et al.^8^

**Exclusion criteria**: pregnancy, breastfeeding, vegetarian diet, history of hypertension, diabetes, allergies and skin diseases, use of immunosuppressants, previous treatments in the chosen area, and weight gain superior to 5% during the study period.

The initial assessment score used was based on the *Investigator Assessment Skin Laxity Scoring System* (IASLSS) scale by Blyumin‐Karasik et al. (Table 1).^8^

**TABLE 1:** Scoring system for assessing sagging skin

Score	Description
0	Toned, firm skin with smooth skin surface texture
1	Slightly smooth, slightly toned skin with smooth skin surface texture
2	Moderately loose, slightly wrinkled skin with crackles on the skin surface
3	Very loose without tone, very wrinkled skins with crackles, separating the skin from the subcutaneous tissue
4	Prominent redundancy of skin without tone, well wrinkled with crackles on the surface of the skin

The skin flaccidity severity score in the pre‐treatment phase ranged from 1 to 3, with the distribution of patients described in Table 2. All photographic documentation was performed in two positions: front and back on the right and left sides. Skin thickness measurements were performed using high‐resolution ultrasound with the Logic E‐R7 version 9.2.1 (GE) device.

**TABLE 2:** Distribution of patients according to severity score

Score	0	1	2	3	4
Number of patients	0	11	10	5	0

**Product preparation**: The Rennova Elleva® bottle, containing 210 mg of PLLA powder, 132 mg of carboxymethylcellulose and 178 mg of pyrogenic mannitol, was reconstituted with 16 ml of sterilized distilled water for injection 1 h before the procedure, through six cycles, consisting in 1 min of vigorous shaking followed by 10 min for reposing, obtaining a translucent and homogeneous suspension. At the time of each application, 0.5 ml of 2% lidocaine without vasoconstrictor and 2 ml of the previously prepared suspension were aspirated in a 3 ml syringe (2.5 ml/syringe), with light homogenization in the syringe to obtain a uniform suspension, making a 20 ml of final volume and a concentration of 10.5 mg/ml of the active ingredient.

**FIGURE 1:**
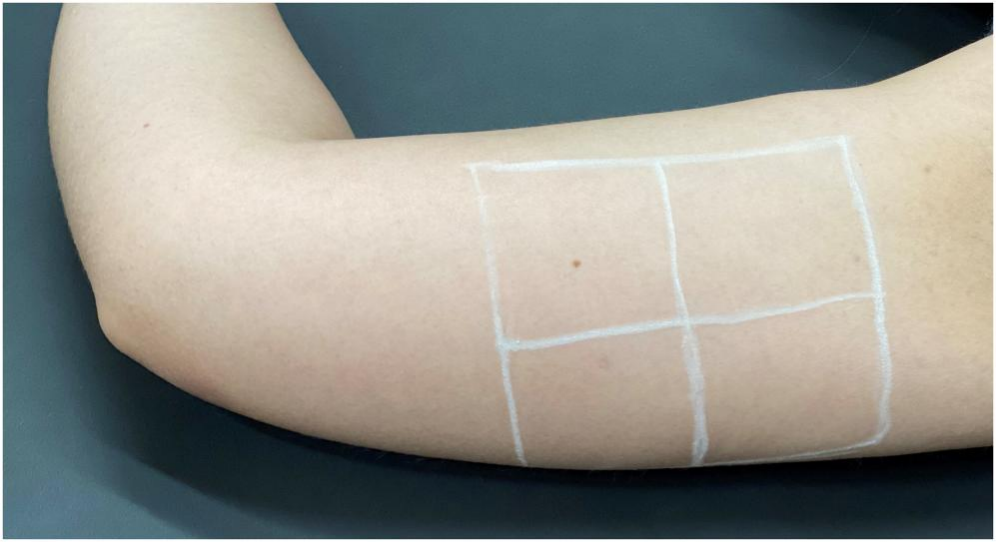
Marking the skin to be treated

**FIGURE 2:**
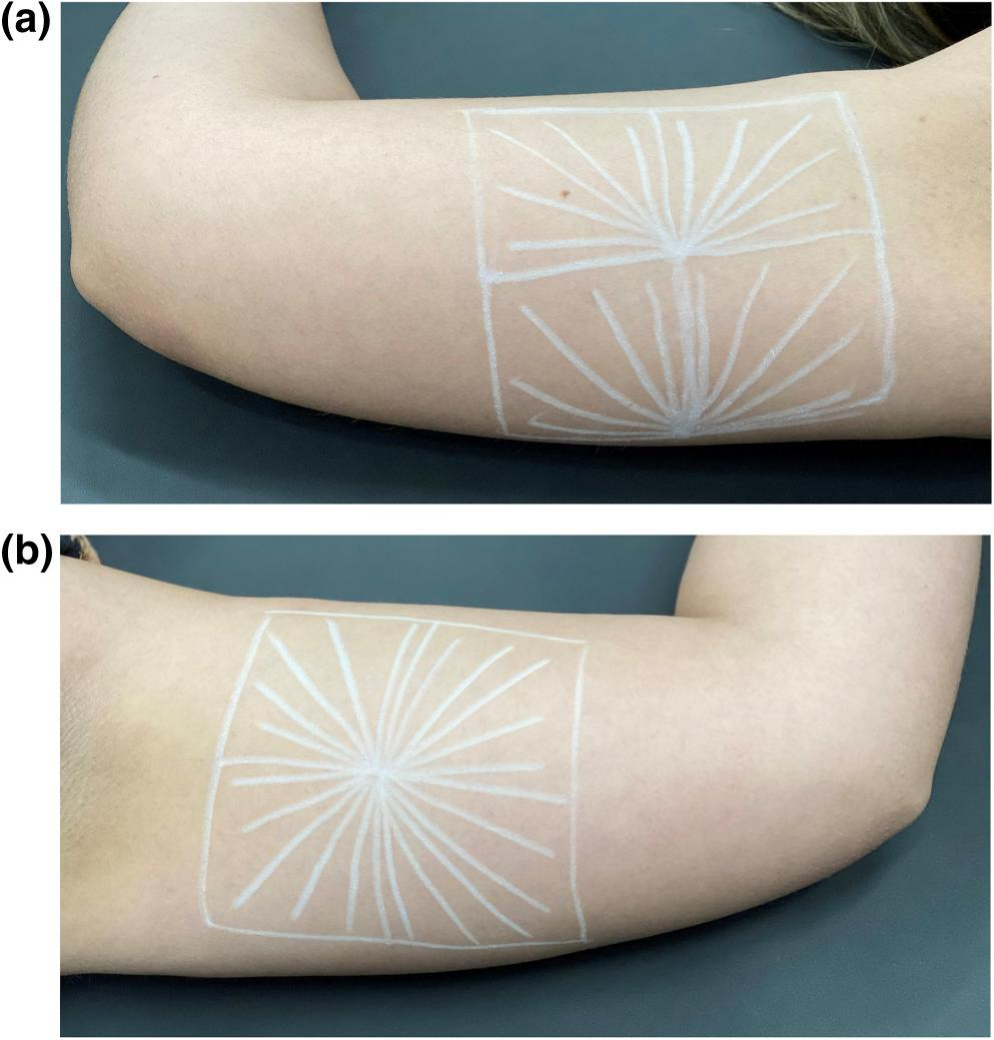
(a,b) Planning the application of the skin to be treated

**Applications**: Applications were performed under local anaesthesia with an anaesthetic cream that united lidocaine and tetracaine. The skin of the inner areas of the arms was marked with four frames of approximately 5 cm,^2^ according to Figure 1. The patients received four syringes containing 2.5 ml of the dispersion with PLLA associated with lidocaine, in the medial area of each arm, totaling the volume of 20 ml in the treatment of both arms per session. The dispersion was injected into the superficial hypodermis using a 22G microcannula, with an entry point at the intersection of the two lateral frames, using a total of two orifices (Figure 2a,b). Five linear back injections of 0.05 ml of the dispersion were performed in each frame, making up 2.5 ml per frame and a total of 10 ml of the dispersion in each arm. Two applications were performed in the medial area of the arms bilaterally with an interval of 45 days between them, using a bottle of PLLA in each application.

After the applications, vigorous local massage was performed for about 5 min and the patients were instructed to vigorously massage the treated area five times a day for 5 min during 5 days.

## 3 RESULTS

**Evaluation**: Forty‐five days after the second application (D90), patients rated treatment tolerability, improvement in skin texture and general satisfaction with treatment, on a scale from 0 (dissatisfaction) to 5 (very high satisfaction), whose results are described in Table 3.

**TABLE 3:** Number of patients according to the score in the parameters: tolerability, improvement in skin texture and general satisfaction

Score	0	1	2	3	4	5
Tolerability	0	0	2	3	10	11
Skin improvement	0	0	0	4	8	14
General satisfaction	0	0	0	3	7	16

After 45 days of the first application of Rennova Elleva® (D45) and 45 days after the second application (D90), skin thickness measurements were performed using the Logic E‐R7 high‐resolution ultrasound exam, version 9.2.1 (GE), comparing with the pre‐treatment period (D0), the values being expressed in percentages of increase in dermal thickness in relation to the initial measurement.

As a complement to the study, a new high‐resolution ultrasound examination was performed 345 days after the last application (9D365) in eight patients.

**FIGURE 3:**
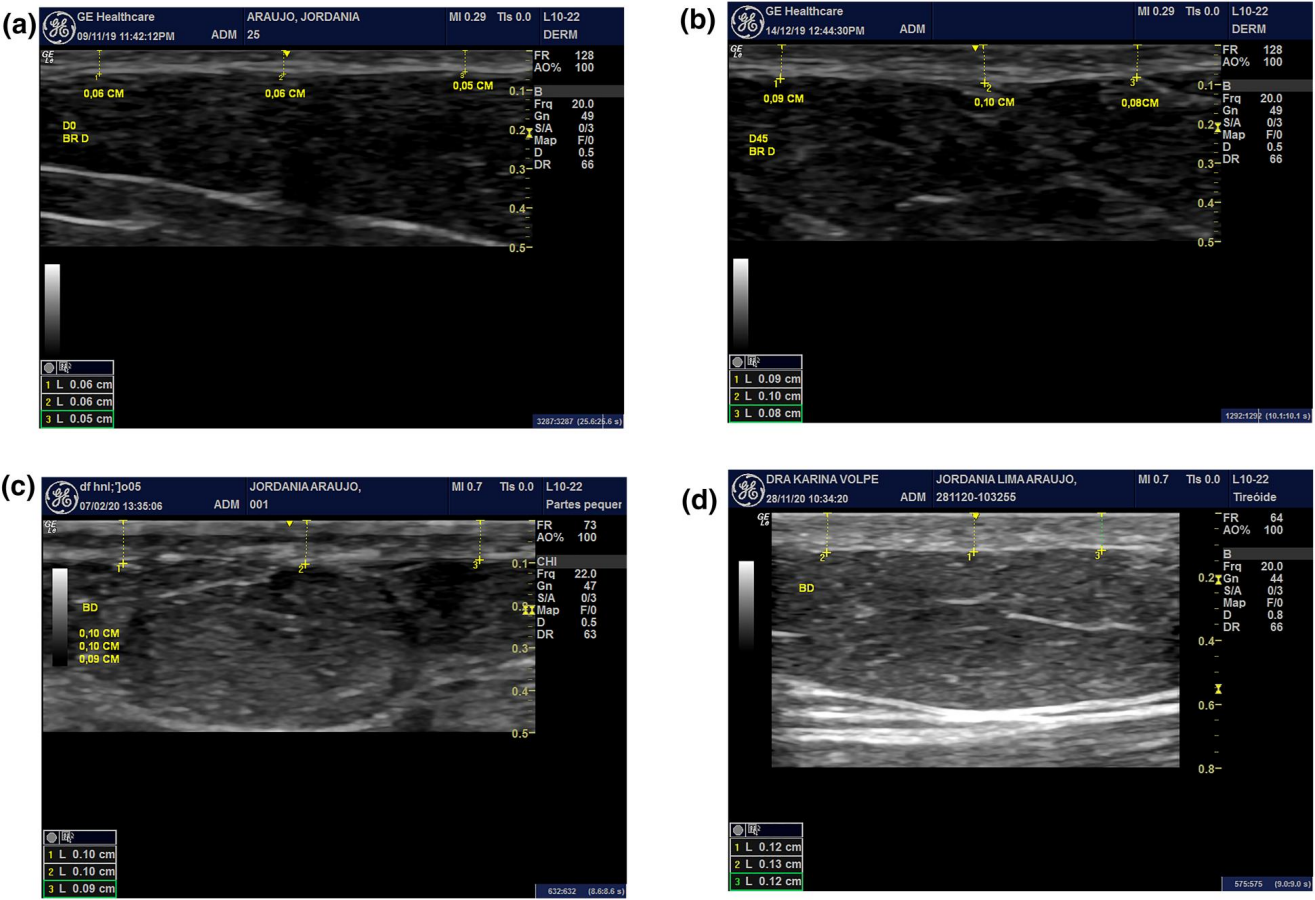
(a) Ultrasounds in the periods A—pre‐treatment (D0). (b) 45 days after the first application (D45). (c) After 45 days of the second application (D90). (d) 365 days after Do (D365)

On high‐resolution ultrasound, the percentages of increase in dermal thickness are described in Table 4 and observed in Figure 3a–d.

**TABLE 4:** Number of patients X percentage values of thickness increase

Days	Number of patients	Minimum value (%)	Maximum value (%)	Average value (%)
D45	18	14	58	29
D90	12	23	70	46
D365	8	26	116	57

**FIGURE 4:**
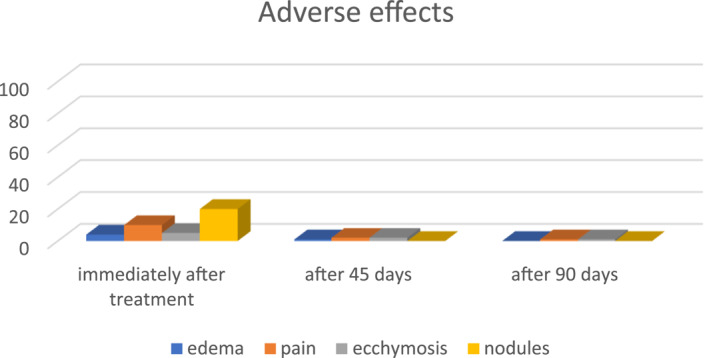
Adverse effects observed: oedema, pain, ecchymosis and presence of nodules; immediately after treatment; after 45 days; after 90 days

The adverse effects observed in each of the two applications were small bruises in some points, oedema, pain, and formation of nodules due to product accumulation (Figure 4). Granulomas of foreign body were not observed during the entire study period.

## 4 DISCUSSION

New and innovative treatments for facial ageing are constantly evolving, but few studies have been published on the treatment of body flaccidity.^7^

Since skin flaccidity is provoked by changes in the collagen and elastic fibres of the dermis and in the fibrous septa of the hypodermis,^8^, ^9^ treatments based on neocollagenesis should offer better results.

Among these treatments we have poly‐l‐lactic acid, whose mechanism of action is to induce a local and gradual reaction that leads to the recovery of the collagen network, which changes during the ageing or stretching process. Once injected, PLLA promotes a subclinical local inflammatory reaction shortly after application, recruiting monocytes, macrophages, and fibroblasts. New collagen begins to form a month after application and continues to build up over a period of 9 months to a year. In the sixth month many PLLA particles become porous, surrounded by macrophages. It is then hydrolyzed into lactic acid monomers and eliminated, leaving an increased deposition of collagen produced by fibroblasts with a consequent increase in dermal thickness, but without evidence of fibrosis. This fibroplasia will determine the cosmetic results, increasing the tissue volume in a gradual and progressive way, which can lead to the recovery of the collagen net and to the improvement of skin flaccidity over the months.^4^, ^5^, ^6^ Results may not be evident for weeks after application, so it is important to wait for the biological reaction to occur between each application to avoid overcorrection.^10^ For the face, the articles recommend 2 to 4 applications with intervals of 30–60 days between them.^6^ Its mechanism of action has important implications for how the product should be applied, how to enhance its results and how to avoid adverse effects.^6^

It is important to emphasize that the final result in the treatment with PLLA depends on many factors, including the amount of product used, the patient's age, the quality of the treated tissue and its collagen stimulation capacity, which varies from patient to patient.^4^, ^6^, ^9^

Patients who obtained a less evident result would probably benefit from a higher number of applications, which was not the objective proposed in this study, as two applications were chosen with intervals of 45 days between them and the pre‐treatment evaluations (D0) and 45 days after the second application (D90), regardless of the factors described here, according to studies by Cunha^11^ and Coimbra,^12^ which stipulated this lapse as the one with the best collagen result in this type of treatment protocol.

The evaluation of the increase in dermal thickness 365 days after the first application was to verify whether the results obtained were sustained or even increased, a fact that was observed in the eight patients evaluated.^13^

The treatment was well tolerated and the response to treatment was in varying degrees and not proportional to the initial classification. According to all patients, there was an improvement in the general appearance of the skin, with a high degree of satisfaction with the treatment. One data observed was that all patients obtained clinical improvement, but the degree of satisfaction was superior to the objective improvement. We must remark that the adverse effects observed are those described in the literature and the formation of early nodules healed spontaneously with no need of intervention.

## 5 CONCLUSION

The application of PLLA to improve facial skin flaccidity is already well known, but there are few reports on its use in the body. In this study we describe a treatment technique to avoid the flaccidity of the inner area of the arms through the subdermal application of Rennova Elleva®, with very promising results.

## AUTHOR CONTRIBUTIONS

**Marisa Gonzaga da Cunha**: Methodology (equal); Supervision (equal); Writing – original draft (lead). **Felipe Mattos Ferregutti**: Investigation (equal). **Amanda Cristina Bernardo**: Investigation (equal). **Pedro Ivo Romani**: Investigation (equal). **Carolina Nascimento**: Validation (equal). **Rogerio Ruiz**: Conceptualization (lead); Methodology (lead); Resources (lead); Supervision (lead); Writing – review & editing (lead).

## CONFLICT OF INTEREST

The authors declare that there is no conflict of interest that could be perceived as prejudicing the impartiality of the research reported.

## ETHICS STATEMENT

CAAE 46120821.6.0000.5373.

## Data Availability

Data openly available in a public repository that does not issue DOIs. The data supporting the findings of this study are available openly on behalf of ORCID 0000‐0002‐4186‐0643 URL: Marisa Gonzaga da Cunha (0000‐0002‐4186‐0643) (orcid.org), reference number not applicable.
